# Disability and poverty – Reflections on research experiences in Africa and beyond

**DOI:** 10.4102/ajod.v2i1.31

**Published:** 2013-08-13

**Authors:** Arne H. Eide, Benedicte Ingstad

**Affiliations:** 1 SINTEF Technology and Society, Oslo, Norway; 2 Department of Community Medicine, University of Oslo, Norway

## Abstract

**Background:**

Whilst broadly agreed in the literature that disability and poverty are closely interlinked, the empirical basis for this knowledge is relatively weak.

**Objectives:**

To describe and discuss the current state of knowledge and to suggest the need for further generation of knowledge on disability and poverty.

**Method:**

Two recent attempts at statistically analysing the situation for disabled people and a series of qualitative studies on disability and poverty are applied in a discussion on the state of current knowledge.

**Results:**

Firstly, the surveys confirm substantial gaps in access to services, and a systematic pattern of lower levels of living amongst individuals with disability as compared to non-disabled. Existing surveys are however not originally set up to study the disability – poverty relationship and thus have some important limitations. Secondly, the qualitative studies have shown the relevance of cultural, political and structural phenomena in relation to poverty and disability, but also the complexity and the contextual character of these forces that may sometimes provide or create opportunities either at the individual or the collective level. Whilst not establishing evidence as such, the qualitative studies contribute to illustrating some of the mechanisms that bring individuals with disability into poverty and keep them there.

**Conclusions:**

A longitudinal design including both quantitative and qualitative methods and based on the current conceptual understanding of both disability and poverty is suggested to pursue further knowledge generation on the relationship between disability and poverty.

## Introduction

It is widely accepted amongst activists, researchers and others that disability and poverty are ‘dynamic and intricately linked phenomena’ (Mitra, Posarac & Vick 2011:1). A rationale for the relationship has been established, with the article by Yeo and Moore (2003) being a key source of reference. Disability leads to poverty through a number of exclusion processes, whilst poverty is a threat to daily life activities, social participation and health, and consequently creates disabling conditions and disability. In his conceptual review of disability and poverty, Palmer (2012) found strong links between poverty and disability regardless of the definition of poverty. Even though research on the relationship is on the increase, it remains limited in low-income countries in particular. Whilst the World Disability Report (WHO 2011) refers to a range of different studies across the world, there is a lack of good data to demonstrate to what extent individuals with disabilities are poorer in different contexts, the diversity amongst individuals with disability, and the mechanisms underlying the ‘vicious circle of disability and poverty’ as to how this plays out in different contexts (Mitra *et al*. 2011; Yeo & Moore 2003:572). It is not necessarily the case, for instance, that the relationship between disability and poverty is the same, or for that matter has the same strength or relevance, in contexts where everyone is poor as compared to more socio-economically differentiated contexts. This article draws on some important experiences in research on disability and poverty in low-income contexts over the last 10 years, and uses these experiences to discuss the state of knowledge and to point towards further research needs.

The conceptual development over the last 10–20 years has expanded the understanding of both disability and poverty. The International Classification of Functioning, Disability and Health (ICF) (WHO 2001) in this regard represents an important milestone in combining a social and a medical model on disability and shifting the balance from bodily functioning to social participation as an outcome of the meeting between an individual and his or her context. This is confirmed through the United Nations Convention on the Rights of Disabled People (CRPD) (UN 2008) as well as the World Report on Disability (WHO 2011), putting disability clearly into a human rights perspective, which is directly relevant for poverty alleviation efforts.

With regards to poverty, a multidimensional understanding emerged in the 1990s through a World Bank study by Narayan (2000), and Spicker (2007) later used the same study to argue for three different definitions of poverty: the basic needs approach, the capability approach, and the economic resources approach. An important, authoritative presentation in this regard was given by Mr James D. Wolfensohn, the former President of the World Bank, in 2004, stating that:

‘Even the understanding of poverty has broadened from a narrow focus on income and consumption to a multidimensional notion of education, health, social and political participation, personal security and freedom, environmental quality …’ (Wolfensohn & Bourguignon 2004:3)

The Millennium Development Goals (MDGs) are a unified set of development goals to address the needs of the world’s poorest (UN 2000). Whilst the MDGs have been heavily criticised for not including disabled people, an increasing recognition of the need for particularly targeting people with disability in poverty alleviation has been demonstrated during recent years, including UN efforts to implement a reporting and monitoring system for disability and the MDGs (UN 2009, 2011) and the High-Level Meeting on disability and development during the 67th session of the UN General Assembly in 2012. The monitoring report treats the disability-poverty relationship explicitly and refers to a ‘feedback loop’ with ‘disability being both a cause and a consequence of poverty’ (UN 2011:7). The increasing recognition of disability as a development issue and as a key element in reaching the MDGs indicates a positive albeit overdue development.

The lack of research on disability and poverty may be partly explained by the fact that both disability and poverty are contested concepts that have undergone important development over the last 10–20 years. Disability, for instance, is referred to as ‘an evolving concept’ in the CRPD (UN 2008: Preamble, point d), and the field of poverty clearly has competing definitions. The situation also reflects the general lack of disability research in poor countries, however, and it may be taken as evidence for the challenges of mainstreaming disability in development. There is limited evidence for the specific manifestations of differences in life conditions between disabled and non-disabled with regards to poverty components, and there are large gaps with regards to how disability leads to poverty and vice versa. Disability statistics, which are potentially powerful both in demonstrating differences and in analysing mechanisms for the relationship between poverty and disability, are far from robust or comparable globally (Eide & Loeb 2006). Whilst important progress has been made with regards to design development and standardisation of disability measures, in particular through the work by the Washington Group on Disability Statistics (Madans, Loeb & Altman 2011), there are still substantial challenges in research on disability and poverty.

This article will explain and discuss first the contributions of two recent attempts at statistically analysing the situation for disabled people and thereafter a series of qualitative studies on disability and poverty. The purpose is not to do a comprehensive literature review on the subject matter, but rather to apply these recent research contributions to a discussion on the direction and content of research on disability and poverty.

## The survey approach to disability and poverty

### The SINTEF southern Africa survey

Between 2002 and 2012, the Foundation for Scientific and Technological Research (SINTEF) carried out national, representative studies on living conditions amongst people with disabilities in collaboration with the Southern Africa Federation of the Disabled (SAFOD), the Norwegian Federation of Organisations of the Disabled (FFO), national universities, national affiliates of SAFOD, central statistical offices, relevant ministries and other key stakeholders in seven countries in the southern African region (Eide & Jele 2011; Eide & Kamaleri 2009; Eide & Loeb 2006; Eide *et al*. 2003; Eide, Van Rooy & Loeb 2003; Kamaleri & Eide 2010; Loeb & Eide 2004;). All studies are cross-sectional, based on the respective national sampling frames, and a representative sample was ensured through collaboration with the central statistical office in each country. Small geographical units (Enumeration Areas) across each country were sampled, and a full listing of all households and individuals within the sampled areas was carried out, including application of the Washington Group on Disability Statistics’ six screening questions (Madans *et al*. 2011). The team of interviewers later re-visited the households with at least one disabled member and interviewed the head of the household and the individual with a disability. In the later studies, a control sample was included, largely by interviewing the head of the household and another matched household member. The questionnaires applied in these surveys were based on previous studies carried out in the region on the level of living and on poverty, and a comprehensive process involving a range of stakeholders and in particular people with disabilities and their organisations.

These studies have together established a unique regional database and a baseline with comprehensive statistical information on the situation amongst individuals with disability and households with disabled members. The later studies with a control sample reveal a largely consistent pattern of differences between individuals and households with and without disability, in rural as well as in urban areas. All studies demonstrate substantial gaps in services, for instance, assistive technology, with nearly half of those who need a device not having access to one. Key indicators on education, mental and physical health, employment, socio-economic status, access to information, social participation, et cetera all point in the same direction: there are substantial gaps in services to disabled people, disability is associated with a lower level of living when compared to non-disabled persons, women with disabilities are worse off than males, and the rural disabled have a lower level of living than their urban counterparts. Although most of the differences are ‘real’ in a statistical sense, many differences are not very dramatic, however, and there is substantial variation between countries. It may be concluded from these studies, however, that disability is clearly associated with lower levels of living in these poor contexts, but also that we need to revisit the over-simplification inherent in the ‘disability-poverty’ axiom. Reality is, not surprisingly, much more complex, and there are clearly other factors than disability influencing this link.

### The World Health Survey

Another recent statistical analysis on disability and poverty is the work carried out by Mitra, Posarac and Vick (2011), utilising data from the World Health Survey (WHS) carried out by the World Health Organization in 2002–2004. The authors aimed at ‘presenting a snapshot of economic and poverty situation of working-age persons with disabilities and their households in 15 developing countries‘ (Mitra *et al*. 2011:1). Of the countries involved in this study, seven were African, four Asian, and four Latin American.

The WHS was a cross-sectional survey and was implemented in 70 developed and developing countries, with the primary objective being to collect comparable health data across countries. It used a common survey instrument in nationally representative populations with different modules to assess the health of individuals in various domains, health system responsiveness, and household expenditures on health care and living conditions.

In all the countries included in the study by Mitra *et al.* (2011), the WHS followed a stratified sample design with weighting. For each household, one informant responded to a household questionnaire including questions on household expenditure, living conditions, assets and household demographics (size and number of children). In addition, within each household, an individual respondent of 18 years or older was selected randomly. That person then responded to an individual-level questionnaire, including questions about his/her own demographic characteristics, disability and health, employment, and education.

The main message from the study is that disability is significantly associated with multi-dimensional poverty in 11 to 14 of the 15 developing countries included in the analyses:

‘In other words, persons with disabilities are more likely to experience multiple deprivations than persons without disabilities in most countries. This result holds when different multidimensional poverty measures and poverty thresholds are used.‘ (Mitra *et al*. 2011:iv)

### Comparing the surveys

The two studies described here are both unique examples of comprehensive and comparable data across countries, and they are amongst the best quality disability statistics from low-income countries. Whilst the WHS is a global survey, the living conditions studies are regional, and whilst the WHS is relatively narrow with regards to indicators, the living conditions studies a broad range of phenomena that in principle covers all aspects of the ICF. Many aspects of the methodology, including the operationalisation of disability, are different between the two studies. In general though, the main messages are similar: (1) disability is associated with systematic lower scores on the selected indicators when comparing with non-disabled, (2) the association between disability and poverty may disguise a more complex relationship due to contextual differences and the heterogeneous character of the population of individuals with disability.

An overall weakness with these studies and others that have been published is their inability to provide evidence beyond the systematic associations. This, of course, is due to the methodology and the cross-sectional design, and the fact that none of the studies were designed to test the disability–poverty relationship in the first place. These studies can demonstrate patterns of poverty and disability, they can analyse relationships between components of a model on disability and poverty, and they can also test more comprehensive models statistically – all important for building the knowledge on disability and poverty. However, they are not based on a model on disability and poverty and thus are not designed to test the disability–poverty relationship. In order to provide stronger evidence for this relationship, the design should be longitudinal and based on one or more relevant theoretical models. The complexity of the phenomena under discussion and problems in applying a classical experimental design further invite mixed methods.

## Qualitative studies on disability and poverty

Whilst different types of surveys may provide a basis for statistics on disability and poverty, qualitative studies may be useful for contributing to theory building, describing individuals’ interpretation and meaning as well as influencing survey design and the interpretation of results from surveys. Although most studies that have dealt more or less directly with disability and poverty have centred on ways of producing statistical data, a series of relevant qualitative studies has recently been accomplished.

*Disability and poverty* by Eide and Ingstad (2011) comprises eight different qualitative studies and two policy analyses. Ten different countries (and cultures) are represented amongst these chapters. They all aim at contributing to the discourse on disability and poverty in low income contexts. The 10 contributions represent an attempt at a culture-sensitive approach to disability, i.e. an understanding of individuals’ values and interpretations as well as the implications of cultural and structural forces on individuals.

Cultural values and meanings represent established patterns for understanding and reacting to a phenomenon. We can identify established and culturally rooted discriminatory practices that affect individuals with disabilities and their families, for instance, gender imbalance as described by Ingstad, Baider and Grut (2011) in their study from Yemen. Segregation between men and women and male dominance play an important role in making girls and women with an impairment more disadvantaged than boys or men (Ingstad *et al*. 2011:148). More than anyone else, poor girls with disability are bound by traditional family patterns and will easily be left isolated, uneducated and unmarried. Paradoxically, as they may face exclusion from the dominant and desired female role, this also creates opportunities for a few girls who, due to a supportive family or other circumstances, may be able to get an education and live an active life because the traditional barriers set up by entering into married life do not apply to them.

Cultural patterns are not static, however, and not even homogenous in a society, and are influenced by collective understanding and practices and by structural and social factors (Ingstad & Whyte 1995). Whilst poverty is largely the result of structural and often international or global forces, a situation of permanent poverty will affect social relations as well as attitudes and, over time, how cultural beliefs and thus also how individuals with disabilities are treated. As described by Grut, Olenja and Ingstad (2011), discrimination against disabled people may easily be seen as a negative cultural practice, whilst another explanation may be that it is simply a forced reaction to poverty, largely a mechanism of survival or absence of options. Hansen and Sait (2011), on the other hand, describe a situation where collective efforts and solidarity contribute to change people’s understanding and thus challenge the political and structural levels in society. This contribution accentuates the potential for human beings even under dire conditions to be able to influence their own situation and challenge dominating forces through collective action. It counters a perspective on disabled people living in poverty as victims that are themselves to blame for discriminatory practices. It is possible that the ability to self-organise, or at least to act concertedly and to establish patterns of meanings that, in the case of South Africa (Hansen & Sait 2011), react against social injustice, is a key ingredient that distinguishes between these two cultural contexts.

The distinction between explaining discrimination and negligence of the needs of disabled people by culture rather than poverty has direct bearings on how researchers, policy makers and other groups external to the situation perceive possibilities for breaking the poverty-disability circle. Emphasising culture may easily lead to inaction, as this is often regarded as a stable phenomenon or at least slowly changing over generations, and representing core values that need to be respected for ethical reasons. Although influence, change and heterogeneity within nations and geographical areas today are seen as key aspects of culture, even in a globalised world, patterns of meaning and practices will still be understood as relatively stable or slowly evolving, and sometimes even reinforced as social reactions to external influence (Friedman 1994). It is again an interesting paradox that intervention at the individual level, i.e. in practice easily implying ‘blaming the individual’, is a preferred level of explanation and action, whilst criticising and attempting to change cultural practice is seen as much more controversial and largely avoided.

The structural level is another obvious level for explaining the persistent relationship between disability and poverty. Muderedzi and Ingstad (2011) describe and analyse how political and structural forces violating basic human rights in Zimbabwe are a direct cause of persistent poverty, with dire consequences particularly for children with disability. One of the most promising theoretical approaches to analysing links between disability and poverty is the introduction of the concepts of ‘social suffering’ and ‘structural violence’ (Farmer 2004; Kleinman *et al*. 1997). Social suffering is imposed on people by conditions outside their control, and can be political, economic, ecological and others. Structural violence plays out where some social structure or social institution purportedly harms people by preventing them from meeting their basic needs, i.e. the violence of everyday life that causes social suffering. By seeing suffering as socially induced, the blame and guilt are placed on the outside forces rather than on the individuals and their families.

Reflecting on the consequences for disabled people of political and structural forces, it may be argued that without putting the needs of individuals with disabilities in the forefront, there is a high risk for maintaining the disability–poverty relationship even if this was not intentional and even in cases where the intention was to alleviate poverty. The voices of the poorest of the poor are easily sidelined, even when they are crucial in combating poverty (Wolfensohn & Bourguignon 2004). Muyinda and Whyte (2011) for instance demonstrate that the exclusion and/or marginalisation of disabled people in essential service development in Uganda results in the needs of disabled people not being met, and consequently contributes to driving individuals and families further into permanent poverty. Attributing the relationship between poverty and disability to social and structural forces underlines the relevance of the political level for breaking the disability-poverty circle.

As demonstrated by Sagli and Fjell (2011), increased political interest for disability policy and development of health and rehabilitation services has not been able to provide necessary services for the rural population in China. A market economy, urban and gender bias combined with the particular political structures of a one-party state has produced a situation whereby services are provided for the most able-bodied of the male, urban disabled, whilst the poor, rural disabled are hit by increasing costs and inadequate health services, even in a situation of rapid economic growth. Likewise, the analyses of policies and instruments in Malawi and Uganda by Wazakili *et al*. (2011) reveals that a disability perspective is easily sidelined in poverty reduction efforts if not specifically incorporated in the process. The contradiction between the policy level and the reality of disabled people living in poverty is further demonstrated in the study by Hansen and Sait (2011) in South Africa, where the introduction of a medically and individually based disability grant conflicts with culturally based solidarity and understanding of disability. These and other examples illustrate very clearly that mechanisms are needed that ensure that the voices of disabled people are heard and acted on, and that a twin-track approach (DFID 2004) combining specifically targeting individuals with disabilities with mainstreaming disability into general poverty alleviation programmes is necessary.

Some authors (Hansen & Sait 2011; Husum & Edvardsen 2011) challenge the very distinction between disability and poverty – *poverty is disability*. With the broadening of the understanding of both concepts, overlap between the concepts and possibly some form of convergence is emerging. Consequently, combating poverty equals the reduction of disabling mechanisms. This may be a very fruitful and not least politically powerful perspective in contexts where poverty is endemic and the consequences of poverty are particularly severe for individuals with disabilities and their families. The view is further interesting in relation to the MDGs and the efforts of the international community to eradicate poverty. A possible consequence of such a viewpoint is found in the UN Monitoring Report (UN 2011:7) which states that ‘A growing body of research now shows that the most pressing issue faced by millions of persons with disabilities worldwide is not their disability but rather poverty. Much of this poverty is the direct and indirect result of exclusion and marginalization of persons with disabilities due to stigma and prejudice about disability’. It is however recognised in the report that the links between disability and poverty are poorly understood, but also that they are more complex and nuanced than previously anticipated.

## Further reflections

The review of recent surveys and qualitative studies on disability and poverty has provided some insights that may contribute to the research field. Firstly, the surveys confirm substantial gaps in access to services, and a systematic pattern of lower levels of living amongst individuals with disability as compared to non-disabled. Because of the design, however, they do have some important limitations. Longitudinal designs based on theoretical models on disability and poverty are suggested as a necessary next step to provide stronger evidence for the mechanisms behind the overrepresentation of individuals with disability amongst the poor. The qualitative studies have shown the relevance of cultural, political and structural phenomena in relation to poverty and disability, but also the complexity and the contextual character of these forces that may sometimes provide or create opportunities either at the individual or the collective level. Whilst not establishing evidence as such, the qualitative studies contribute to illustrating some of the mechanisms that bring individuals with disability into poverty and keep them there.

The association between disability and poverty is real in low-income contexts, but it may not always be as marked as we tend to think, and it may sometimes not play out at all. A disability status gives challenges but, for some, also opportunities that he or she would not have got without being disabled. Our point is that we should be able to have two things in mind at the same time: disability and poverty are linked, but also that many individuals with disability manage – and that individuals with disability as a group are just as heterogeneous as other population groups, also when it comes to economic, social and political inclusion. Endemic poverty in many ways creates the negative conditions that affect all, whilst the consequences of disability may be seen as more fluid and depend on a whole range of factors that can either be barriers or facilitators for inclusion and participation.

Whilst the MDGs have been heavily criticised for not including disability (Albert 2006), it may be argued that it is mostly about poverty in the sense that eradicating poverty will also imply preventing disability, alleviating the consequences of disability, and eradicate disabling conditions. This however, may be questioned simply by observing the situation globally. In many societies at different levels of welfare and economic development, there is a persistent pattern of disabled people being poorer and less engaged and less able to participate in society, for example in employment and education, as compared to non-disabled. Loeb *et al*. (2008), for instance, showed that the relatively generous disability grant in South Africa did remove the economic (income) differences between households with and without disabled members, but that differences remained with regards to other elements in a broader conception of poverty.

Bringing people out of poverty will thus not in itself eradicate disability and disabling conditions, regardless of the level of understanding of disability. Many of the mechanisms that side line individuals with disabilities in society are at work in developed welfare states as well as in poverty-stricken countries. This implies primarily that disability, discriminatory practice, cultural beliefs, environmental barriers, lack of equitable basic services, etc., are all factors that need to be dealt with or utilised in poverty alleviation efforts in order to ensure that people with disabilities benefit in an equitable manner. Otherwise, the risk is that a segment of society, i.e. individuals with disabilities and other vulnerable groups, will remain in poverty whilst a successful reduction in the poverty rate is celebrated.

Individualisation of disability, as we find in the Western/European-dominated discourse on disability (Mollow 2004), has its evident limitations when the main problems are structural and political. In this perspective it is interesting that recent development of the conceptual understanding of disability, has, in fact, incorporated social and political structures (environment). Phenomena at this level are thus accepted as being central parts of the disablement process. Whilst cultural, political and structural phenomena clearly can cause poverty and disability, we do not, however, understand these contributions as presenting arguments against the relevance of the individual level. Rather, in poverty-stricken contexts, political and structural changes will be cardinal in allowing people to live their lives in dignity and to be able to fulfil their potential, contributing to their families and to the community.

The different levels of explanation are intertwined, and it would be a mistake to discard individuals’ own efforts. Individuals with disabilities living in poverty do struggle to survive and to make the best out of their situation – and there are encouraging examples of individuals who have used their disability as a resource for themselves and for others in the community. The distinction between the political/structural level and the individual level is a reality, however, and many individuals with disabilities are born into or brought into poverty by forces outside themselves and their families. Bearing in mind the dangers of victimisation and defeatism, it is nevertheless evident that structural, political and even cultural changes are crucial for breaking the poverty disability circle. It is particularly important to underline this as the understanding of disability as well as interventions often centre around the individual. Even the ICF, with its attempt at incorporating environmental or social factors, basically represents an individual understanding, at least if this is not challenged and the environmental aspects are not further developed and strategically utilised.

The different levels of explanation, which is also where the keys for breaking the poverty-disability circle may be found, cannot be viewed separately from each other. Policy changes with the best of intentions may fail or even be counterproductive if people’s cultural beliefs, structural barriers or policy shortcomings, are not considered as playing key roles. Resourceful individuals and communities may fail in their countermeasures if overcome by forces of structural violence. Further, whilst there are clearly general knowledge and experiences that can contribute to understand disability and poverty as a global phenomenon, contexts are different and require separate analyses and unique solutions.

A valuable challenge to established and largely Western-dominated thinking around disability and poverty is found in the distinction between individualised and political or structural explanations, but this distinction should better inform research to avoid individual bias and to ensure that research is based on an understanding of the intertwined relationship between the two levels. Including disabled people and their representatives or advocates in the policy process is not only correct in a democratic and human rights perspective, it is also crucial for finding the right solutions. Finally, due to the complexity of the disability–poverty relationship, it will be necessary to draw on a range of methods, including longitudinal survey research to test active mechanisms statistically with qualitative approaches revealing meanings and cultural values of significance for both social and structural phenomena as well as individual interpretation and choices.

Whilst disabled people in poor contexts are and have been deprived of basic services, the inclusion of individuals with disabilities will in many instances be a challenge due to lack of education, experience and not least due to weak organisations. It is of importance to recognise this and to put long-term capacity building in place. However, individuals with disabilities have struggled, survived and managed to influence the international discourse on disability and poverty under very difficult conditions, bringing evidence to the fact that they also represent a tremendous resource that can be used to improve the situation for the poorest of the poor. After all, individuals with disabilities are experts on living with disabilities. Without this experience, and without challenging and breaking up established power structures, the fight against poverty will be jeopardised.
